# Editorial: Pulmonary fibrosis and endocrine factors

**DOI:** 10.3389/fendo.2024.1519318

**Published:** 2024-11-13

**Authors:** Pengxiu Cao, Hong-Long (James) Ji, Jianwei Sun

**Affiliations:** ^1^ Ministry of Education Key Laboratory of Molecular and Cellular Biology, Hebei Key Laboratory of Animal Physiology, Biochemistry and Molecular Biology, College of Life Sciences, Hebei Normal University, Hebei Research Center of the Basic Discipline of Cell Biology, Hebei Collaborative Innovation Center for Eco-Environment, Shijiazhuang, Hebei, China; ^2^ Department of Surgery, Burn and Shock Trauma Research Institute (BSTRI), Stritch School of Medicine, Loyola University Chicago, Maywood, IL, United States; ^3^ Yunnan Key Laboratory of Cell Metabolism and Diseases, State Key Laboratory for Conservation and Utilization of Bio-Resources in Yunnan, Center for Life Sciences, School of Life Sciences, Yunnan University, Kunming, China

**Keywords:** pulmonary fibrosis, apolipoprotein B, glycolysis, diabetes, complement, vitamin D

## Background

Pulmonary fibrosis (PF) is a complex and highly heterogeneous disease characterized by the accumulation of stiffened extracellular matrix in lung tissue, the replacement of normal lung tissue with fibrotic tissue, and the progressive loss of lung function. PF is induced by genetic, environmental, and inflammatory factors and is associated with metabolic disorders. Extracellular bioactive mediators, such as endocrine factors and metabolites, play critical roles in the progression of PF. For instance, iron promotes PF by activating fibroblasts (1). Animal models have also demonstrated that thyroid hormone, brain-derived neurotrophic factor, and atrial natriuretic peptide regulate PF (2–4). Focusing treatment on dysregulated endocrine factors rather than inhibiting downstream fibrosis markers may be more effective. In addition, supplementing endogenous cytokines or metabolites theoretically results in fewer side effects than exogenous drugs. This Research Topic, “*Pulmonary Fibrosis and Endocrine Factors*”, aims to define the endogenous factors associated with pulmonary fibrosis in order to facilitate the discovery of novel and effective therapeutic strategies with fewer side effects.

## Summary of published studies

This Research Topic addresses the roles of lipid metabolism, glucose metabolism, complement pathway, and serum biomarkers in PF or cystic fibrosis (CF). It also investigates the association of vitamin D metabolism-related single nucleotide polymorphisms with the risk of chronic obstructive pulmonary disease (COPD).

We highlighted two pioneering studies examining the relationship between lipid metabolism and fibrosis. Jiang et al. utilized a Mendelian randomization approach to investigate the influence of endocrine and metabolic factors on the risk of idiopathic pulmonary fibrosis (IPF). Based on data from a genome-wide association study, they analyzed 53 endocrine and metabolic traits and identified significant associations between seven of these traits and IPF. Specifically, apolipoprotein B was found to be negatively associated with IPF risk. In addition, factors such as weight, body mass index, whole body fat mass, waist circumference, trunk fat mass, and body fat percentage are positively correlated with IPF risk. These results were validated by multiple sensitivity analyses, and no significant horizontal pleiotropy or heterogeneity was found, further enhancing the reliability of the results.

The article by Qi et al. provided a systematic evaluation of the relationship between leptin and CF. Through a meta-analysis of 919 CF patients and 397 controls with serum/plasma leptin concentrations appearing in 14 studies, it was found that there was no significant difference in leptin levels between CF patients and healthy individuals. However, subgroup analyses considering factors such as gender, sample testing methods, age, and study design indicated that female CF patients had higher leptin concentrations than their male counterparts, and healthy males had lower leptin levels than healthy females. The study also observed a positive correlation between serum/plasma leptin levels and fat mass, and BMI as well. These results pave the way for further exploration of the regulatory role of leptin in PF.

This Research Topic also delves into the complex relationship between glucose metabolism and PF. Wang et al. conducted a systematic review of the roles of key glycolytic enzymes in IPF. They reported increased expression of several enzymes, including hexokinase 2, 6-phosphofructo-2-kinase/fructose-2,6-bisphosphatase 3, and lactate dehydrogenase, in both IPF patients and murine pulmonary fibrosis models. These enzymes significantly impact the progression of PF by promoting the expression of fibrotic markers. In addition, lactate enhanced fibroblast differentiation by activating macrophages and triggering TGF-β. The study emphasized several critical regulatory factors that mediate interactions between PF and glucose metabolism, such as TGF-β, IL-1β, HIF-1α, NOX4, ATF4, and AGEs. The study also pointed to hyperglycemia as a potential contributor to PF. Further, in another study, Mzimela et al. evaluated the impact of type 2 diabetes mellitus (T2DM) on pulmonary vascular function and PF development. In T2DM patients, hyperglycemia and dyslipidemia induce pulmonary vascular dysfunction, including endothelial dysfunction, vascular remodeling, and abnormal pulmonary vascular tone and responsiveness. Diabetes-associated hyperglycemia and dyslipidemia enhance the production of reactive oxygen species and pro-inflammatory cytokines, triggering inflammatory responses and cellular damage in lung tissue. Prediabetes also exhibits similar adverse effects on lung health; resolving lung disease in prediabetic individuals may prevent progression to overt T2DM. This underscores the importance of early detection and intervention in preventing T2DM and its associated pulmonary complications.

This Research Topic investigates the roles of the immune system and vascular factors in PF. Sikkeland et al. examined the involvement of the terminal C5-C9 complement pathway in IPF. Through high-resolution proteomics analysis of broncho-alveolar lavage (BAL) from IPF patients and the control subjects, the study identified 567 differentially regulated proteins (118 upregulated and 449 downregulated), with the complement and coagulation systems as the most significantly affected pathways. All components of the terminal complement complex (TCC) (C5, C6, C7, C8, and C9) were significantly upregulated in the BAL of IPF patients. Additionally, plasma TCC levels were significantly higher in IPF patients than controls and were significantly correlated with complement factors C3, C8, and C9 in the BAL in IPF patients with elevated plasma TCC.

Zhong and Luo discovered that serum levels of Krebs von den Lungen-6 (KL-6) were positively correlated with the extent of fibrotic damage in idiopathic pulmonary fibrosis interstitial lung disease (IPF-ILD) patients and serum vascular endothelial growth factor (VEGF) levels were associated with disease progression in IPF-ILD. Both KL-6 and VEGF showed significant correlations with partial pressure of arterial oxygen, a measure of pulmonary gas function. Although this study included small size of samples and utilized cross-sectional design, which precludes establishing causality, the findings suggest that KL-6 and VEGF are reliable indicators of pulmonary oxygenation status. These two biomarkers demonstrate high specificity and sensitivity in the diagnosis, monitoring, and prognosis for IPF-ILD.

Additionally, Rojo-Tolosa et al. conducted the first comprehensive study of the impact of 13 vitamin D metabolism-related single nucleotide polymorphisms on the risk of COPD. This retrospective study analyzed genetic data from 152 COPD patients and 456 control subjects. The findings also showed that the AA genotype and A allele of CYP27B1 rs4646536 and CYP2R1 rs10741657 were associated with a significantly increased risk of COPD. In addition, the GATG and AGGT haplotypes correlated with an elevated COPD risk, underlining the importance of genetic variations in the vitamin D metabolic pathway for COPD risk assessment. Although this study did not directly evaluate the impact of vitamin D levels on COPD, it provides new insights into the genetic basis of COPD and points out directions for future research and clinical trials. Meanwhile, this study also provides important reference for IPF related research.

## Conclusions and future directions

These studies have enhanced our understanding of lipid metabolism, glucose metabolism, complement and vascular factors, and the complex role of vitamin D in PF. Lipid metabolism may affect the progression of PF through factors such as apolipoprotein B. In addition, key enzymes and regulatory factors in the glycolysis pathway, complement system, and vascular factors provide new potential targets for treating IPF, opening up new directions for the clinical diagnosis and treatment of PF. Clinically, it is necessary to pay attention to the metabolic status, diabetes history, abnormal vitamin D metabolism, and other genetic factors related to PF patients. To develop targeted personal therapeutic strategies, future research should continue to explore the specific mechanisms of these endocrine and metabolic characteristics with the potential to reveal deeper pathological mechanisms, identify novel therapeutic targets, or discover highly sensitive and specific biomarkers for pulmonary fibrosis.
